# Robust spin-orbit torque and spin-galvanic effect at the Fe/GaAs (001) interface at room temperature

**DOI:** 10.1038/ncomms13802

**Published:** 2016-12-13

**Authors:** L. Chen, M. Decker, M. Kronseder, R. Islinger, M. Gmitra, D. Schuh, D. Bougeard, J. Fabian, D. Weiss, C. H. Back

**Affiliations:** 1 Institute of Experimental and Applied Physics, University of Regensburg, 93040 Regensburg, Germany; 2 Institute of Theoretical Physics, University of Regensburg, 93040 Regensburg, Germany

## Abstract

Interfacial spin-orbit torques (SOTs) enable the manipulation of the magnetization through in-plane charge currents, which has drawn increasing attention for spintronic applications. The search for material systems providing efficient SOTs, has been focused on polycrystalline ferromagnetic metal/non-magnetic metal bilayers. In these systems, currents flowing in the non-magnetic layer generate—due to strong spin–orbit interaction—spin currents via the spin Hall effect and induce a torque at the interface to the ferromagnet. Here we report the observation of robust SOT occuring at a single crystalline Fe/GaAs (001) interface at room temperature. We find that the magnitude of the interfacial SOT, caused by the reduced symmetry at the interface, is comparably strong as in ferromagnetic metal/non-magnetic metal systems. The large spin-orbit fields at the interface also enable spin-to-charge current conversion at the interface, known as spin-galvanic effect. The results suggest that single crystalline Fe/GaAs interfaces may enable efficient electrical magnetization manipulation.

## Abstract (web summary)

Interfacial spin-orbit torque allows electrical manipulation of magnetization, but this has been shown mostly in polycrystalline metal bilayers. Here the authors show robust spin-orbit torque in single crystalline Fe/GaAs interface at room temperature, observing conversion between spin and charge current.

In solids with space-inversion asymmetry intrinsic spin-orbit fields (SOFs) emerge. These SOFs can arise from Dresselhaus spin–orbit interaction (SOI) which originates from bulk inversion asymmetry^1^, and/or from Bychkov-Rashba SOI which originates from structure inversion asymmetry^2^. In the limit of small wave vector **k** near the Γ point, both SOIs can be expressed by k-linear terms in the Hamilton operator; that is, *H*_D_=*β*(*σ*_*x*_*k*_*x*_−*σ*_*y*_*k*_*y*_) in case of Dresselhaus SOI and *H*_R_=*α*(*σ*_*x*_*k*_*y*_−*σ*_*y*_*k*_*x*_) in case of Bychkov-Rashba SOI. Here *σ*_*x,y*_ are Pauli spin matrices and *α* and *β* characterize the strength of Rashba and Dresselhaus spin-orbit coupling. Figure 1a,b show the SOFs due to Rashba and Dresselhaus SOI. The most striking property of these SOIs is that spin and carrier momentum are coupled, meaning that a charge current is accompanied by a non-equilibrium spin accumulation^3^,^4^,^5^. The magnitude and orientation of this spin accumulation is controlled by the charge current. It is convenient to quantify this spin accumulation by an effective SOF (eSOF). On the other hand, the inverse process is also applicable: a non-equilibrium spin accumulation drives a charge current^6^. Making use of SOI is thus a promising pathway for future spintronic applications^7^. In fact, it has been demonstrated that SOI can be used to manipulate the magnetization direction by a local current^8^,^9^,^10^,^11^. Thus, establishing a new material system with strong SOI at room temperature is of utmost importance for the realization of practical spin-orbit torque (SOT) devices. Motivated by early work on strained low-temperature (Ga,Mn)As^10^,^11^,^12^,^13^, it has been shown recently that there is sizeable Dresselhaus dominated SOT in the Heusler alloy NiMnSb^14^. Furthermore, electric switching in the antiferromagnet CuMnAs by an internal SOT has been demonstrated at room temperature^15^.

It is well established that single crystalline Fe thin films grow epitaxially on GaAs (001) due to the small lattice mismatch between twice the lattice constant of Fe (2.87 Å) and GaAs (5.65 Å). The Fe/GaAs interface has *C*_2*v*_ symmetry. For this particular interface it has been shown that near the Fermi energy and in the vicinity of the Γ-point the SOFs arising from *C*_2*v*_ symmetry can be expressed as a combination of Dresselhaus- and Bychkov-Rashba-like SOFs^16^, causing, for example, an anisotropic tunneling magnetoresistance^17^ and a crystalline anisotropic magnetoresistance (AMR) with two-fold symmetry, when the Fe layer is only a few monolayers thick^18^.

Here we demonstrate that a robust SOT exists at the Fe/GaAs interface at room temperature. We find that the dominating eSOF contribution stems from Bychkov-Rashba-like SOI, and importantly, that the magnitude of the eSOF is comparable to the ones in ferromagnetic metal/non-magnetic metal systems^19^,^20^,^21^,^22^. Moreover, we show that the strong SOI at the interface converts a pure spin current, generated by spin pumping^23^,^24^,^25^, into a charge current. This is due to the spin-galvanic effect (SGE)^6^ also called inverse Rashba-Edelstein effect^26^, which is similar to the inverse spin Hall effect (ISHE) in a three dimensional material^7^. Apart from semiconductors it has only been observed at an Ag/Bi interface^26^ and in the three dimensional Rashba-metal GeTe^27^. The simple structure of Fe/GaAs used here excludes any bulk contribution via ISHE since the conducting non-magnetic layer is absent. Our results indicate that a single crystalline Fe/GaAs interface is sufficient for electrical magnetization manipulation.

## Results

### Quantifying eSOFs by spin-orbit ferromagnetic resonance

We determine the strength of the interfacial SOFs by using spin-orbit ferromagnetic resonance (SO-FMR) developed previously to quantify SOFs in the ferromagnetic semiconductor (Ga,Mn)As^12^,^13^. SO-FMR is similar to the spin-torque ferromagnetic resonance (FMR) technique which is used to quantify the spin Hall effect in ferromagnetic metal/non-magnetic metal bilayers^9^. The mechanism is illustrated in Fig. 2a. An alternating in-plane current **j**(*t*) flowing in the Fe stripe generates, due to SOI, a time dependent non-equilibrium spin accumulation at the Fe/GaAs interface. This spin accumulation exerts a torque on the magnetization of the Fe film and can be viewed as a time varying interfacial magnetic field **h**_SO,_ which excites the magnetization dynamics. The precessing magnetization causes a resistance variation *R*(*t*) via the AMR of Fe. Due to mixing of the alternating current and the oscillating resistance, a dc voltage *V* results^28^. Thus, by measuring this dc voltage for different crystallographic directions we can quantitatively determine the effective spin-orbit fields at the Fe/GaAs interface.

**Figure 1:**
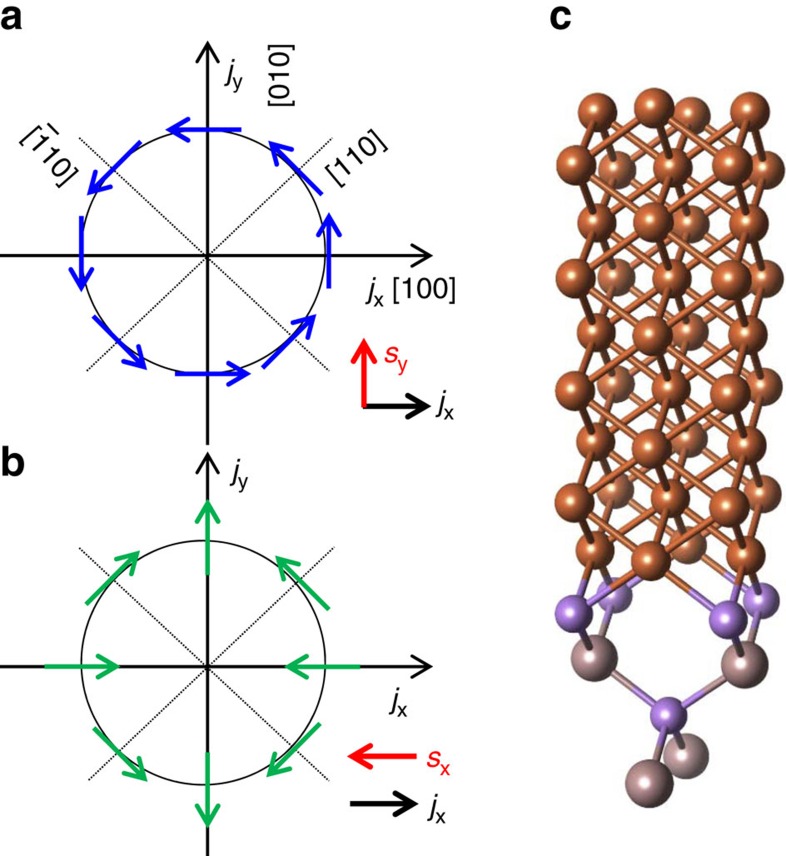
Spin-orbit coupling at the Fe/GaAs interface. Schematic of Rashba (**a**) Dresselhaus (**b**) spin-orbit fields (SOFs) for different crystallographic orientations. Red arrows in **a**,**b** denote the direction of spin accumulation induced by a current flow *j*_x_. (**c**) Atomic structure of the Fe/GaAs (001) spin–orbit interface. Zincblende GaAs exhibits bulk inversion asymmetry (BIA) with *D*_2*d*_ symmetry and adding a single crystalline Fe on top of GaAs further lowers the *D*_2*d*_ symmetry to *C*_2*v*_. The *C*_2*v*_ symmetry results in the Rashba and Dresselhaus SOFs at the Fe/GaAs (001) interface.

**Figure 2:**
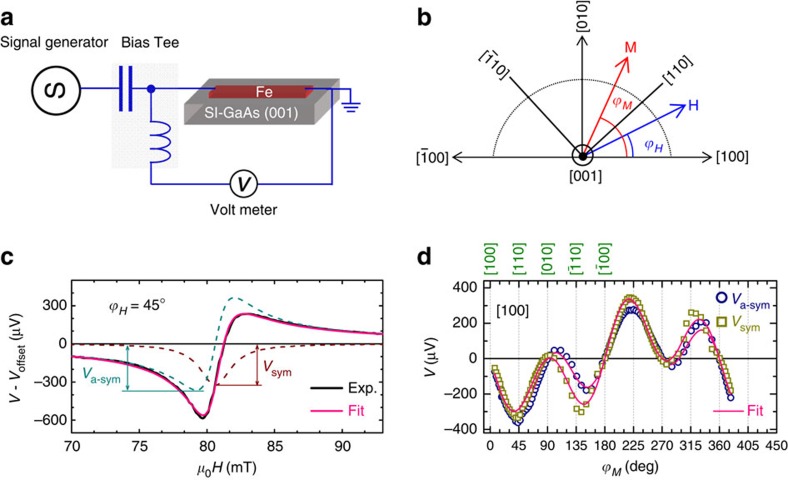
Spin-orbit ferromagnetic resonance measurements. (**a**) Depiction of sample structure and experimental set-up. A microwave current passes through the Bias Tee and to the sample to drive the magnetization dynamics in the Fe film. A rectified dc voltage is detected across the Fe stripe. (**b**) Definition of magnetic-field angle *ϕ*_*H*_ and magnetization angle *ϕ*_*M*_. (**c**) Typical spectrum of the dc voltage *V* obtained at a magnetic-field angle of *ϕ*_*H*_=45°, microwaves frequency of 12 GHz, and temperature of 300 K, where the offset voltage *V*_offset_ is subtracted. (**d**) Dependence of *V*_a-sym_ and *V*_sym_ on the magnetization angle *ϕ*_*M*_ for a [100]-orientated device. The solid lines are fits to equation (1).

The sample used in our study is a 5-nm thick single crystalline Fe film grown by molecular-beam epitaxy (MBE; see Fig. 1c and Methods) on top of a 100 nm thick undoped GaAs buffer layer on a semi-insulating GaAs (001) substrate. Additionally, we grew a polycrystalline Fe reference film on an amorphous SiO_*x*_ substrate. Devices of size, 6.4 × 100.0 μm, oriented along different crystallographic directions, are defined by employing electron-beam lithography and ion beam etching. Figure 2c shows a typical *V* trace (where an offset voltage *V*_offset_, due to thermo-electric effect, has been subtracted) of a [100]-orientated stripe measured at room temperature. To fit the characteristic line shape, we introduce a symmetric (*L*_sym_=Δ*H*^2^/[4(*H*−*H*_R_)^2^+Δ*H*^2^]) and an anti-symmetric Lorentzian (*L*_a-sym_=−4Δ*H*(*H*−*H*_R_)/[4(*H*−*H*_R_)^2^+Δ*H*^2^]), where *H*_R_ is the magnetic field *H* at FMR, Δ*H* the line width (full width at half maximum: FWHM). *V*−*V*_offset_ is fitted by a combination of *L*_sym_ and *L*_a-sym_, *V*_sym_*L*_sym_+*V*_a-sym_*L*_a-sym_, with *V*_sym_ (*V*_a-sym_) the magnitude of the symmetric (anti-symmetric) component of the dc voltage. By fitting we obtain values for *H*_R_, Δ*H, V*_sym_ and *V*_a-sym_. These parameters have been extracted as a function of *ϕ*_*H*_ and for Fe stripes along different crystallographic directions (see Supplementary Figs 3 and 5). The *ϕ*_*H*_-dependence of *H*_R_ and Δ*H* can be well explained by conventional FMR analysis^29^,^30^, from which we obtain the Landé g factor (*g*=2.12), the effective demagnetization field (*μ*_0_*H*_K_=1,750 mT), the biaxial magnetic anisotropy field (*μ*_0_*H*_B_=40 mT), the uniaxial magnetic anisotropy field (*μ*_0_*H*_U_=48 mT), and the damping constant (*α*=0.0036) (see Supplementary Note 3).

For comparison, we also tried to measure dc voltages on polycrystalline Fe films deposited on thermally oxidized Si substrates; however, no characteristic dc voltages can be observed at FMR using the same excitation conditions as for single crystalline films of Fe/GaAs, indicating that the driving force for magnetization dynamics originates from the eSOFs at the interface of single crystalline Fe/GaAs and not from any current induced Oersted field (see Supplementary Note 1).

To quantify the magnitude of the eSOFs, it is necessary to understand the dependence of the dc voltage on *ϕ*_*M*_. By taking a [100]-orientated stripe as an example, the anti-symmetric and symmetric components can be expressed as (for the derivation of other orientations see Supplementary Note 2 and 3)


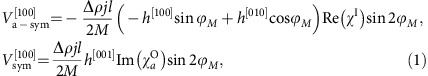


where *h*^[100]^, *h*^[010]^, *h*^[001]^ are the eSOFs along the [100], [010] and [001] directions, Δ*ρ* is the magnitude of AMR, *j* the microwave current density, *l* the length of the device, *M* the magnetization of the Fe film, Im(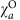) the imaginary part of the off-diagonal component of the magnetic susceptibility due to out-of-plane excitation (*h*^[001]^), and Re(*χ*^I^) the real part of the diagonal component due to in-plane excitation (*h*^[100]^ and *h*^[010]^). Since the dc voltage is induced by FMR, the magnitude of Im(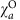) and Re(*χ*^I^) can be calculated by solving the Landau–Lifshits–Gilbert equation^31^, which is anisotropic due to the in-plane magnetic anisotropy as well as the angular variation of the linewidth^30^ (see Supplementary Note 3).

### In-plane effective spin-orbit fields

Figure 2d shows the dependence of *V*_a-sym_ on the magnetization angle *ϕ*_*M*_ for the stripes oriented along the [100] direction. The results can be fitted by a superposition of sin2*ϕ*_*M*_ sin*ϕ*_*M*_ and sin2*ϕ*_*M*_ cos*ϕ*_*M*_, indicating that the driving field contains both Dresselhaus and Bychkov-Rashba contributions (see equation (1)). The magnitude of *μ*_0_*h*^[100]^ (Dresselhaus) and *μ*_0_*h*^[010]^ (Bychkov-Rashba) is determined to be −0.15 and 0.28 mT by using *μ*_0_*M*=1.63 T, Δ*ρ*=7.0 × 10^−10^ Ωm, *l*=100.0 μm and *j*=1.91 × 10^11^ Am^−2^. Here *M* and Δ*ρ* have been determined by separate magnetization and dc transport measurements, and the microwave current *j* is calibrated by thermally shifting the resonance field (see Supplementary Note 5).

For an independent crosscheck, we also show in the Supplementary Note 7 that the Dresselhaus field can be alternatively verified by varying *H*_R_ via a dc current.

**Figure 3:**
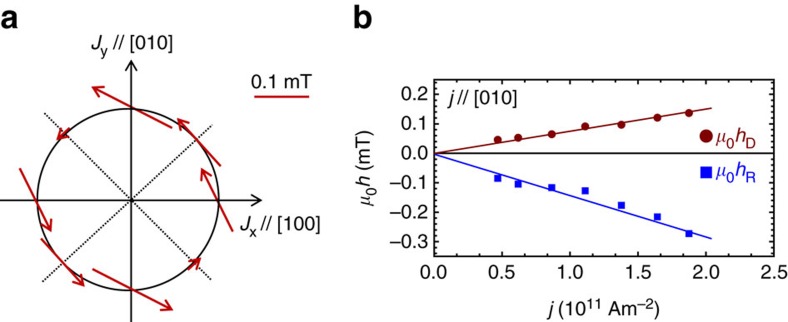
In-plane spin-orbit fields. (**a**) Experimentally determined magnitude and direction of the in-plane spin-orbit fields, which are normalized by a unit current density of 10^11^ Am^−2^. (**b**) Current density dependence of the magnitudes of *μ*_0_*h*_R_ and *μ*_0_*h*_D_ obtained from a [010]-orientated device. From the slope of *μ*_0_*h*_R_ and *μ*_0_*h*_D_, the ratio of *α* and *β*, (*α*/*β*)_in-plane_, is determined to ∼2.0.

In Fig. 3a, we show direction and magnitude of the in-plane effective spin-orbit fields obtained for a current density of 10^11^ Am^−2^ in a polar plot for devices with different orientations. Figure 3a indicates that Bychkov-Rashba is the dominating contribution since the field vectors along [100] and [010] directions align mainly perpendicular to the current direction. For [110] and [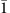
10] directions, the spin-orbit fields are perpendicular to the current as expected for both Rashba and Dresselhaus contribution (Fig. 1a,b). Although the current induced Oersted field may also cause a field contribution perpendicular to the current directions, this effect is expected to be small for two reasons: First, the magnitude of the SOF for the [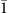
10] direction is ∼5 times smaller than that for the [110] direction for a similar current density (Fig. 3a and Supplementary Fig. 5b), which indicates that the effect is intrinsically related to SOI. Second, the non-detectable dc voltage in Fe/SiO_*x*_ further excludes a sizeable influence of any Oersted field. The different magnitudes of the eSOFs along the [110] and [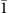
10] directions stem from constructive and destructive superposition of Dresselhaus and Bychkov-Rashba fields (see Fig. 1a,b). In Fig. 3b, we show that the amplitudes of *μ*_0_*h*_R_ and *μ*_0_*h*_D_ in the [010] stripe increase linearly with the current density, indicating that reduction of *M* and Δ*ρ* due to Joule heating is negligibly small in the device. From the slope of *h*_R_ and *h*_D_, the ratio of *α* and *β*, (*α*/*β* )_in-plane_, is determined to be ∼2. We have repeated these measurements for two more sets of devices (eight devices); each set shows similar behaviour.

### Out-of-plane effective spin-orbit fields

**Figure 4:**
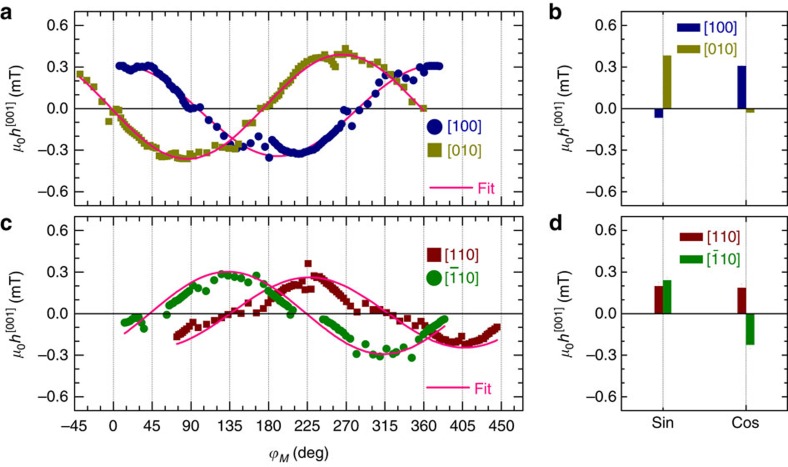
Out-of-plane spin-orbit fields. (**a**) Magnetization angle *ϕ*_*M*_ dependences of the out-of-plane spin-orbit field *μ*_0_*h*^[001]^ for [100] and [010]-orientated devices; the solid lines are fits by equation (2), from which (*α*/*β*)_out-of-plane_ is obtained (see Supplementary Note 8). The fitting coefficients of sin*ϕ*_*M*_ and cos*ϕ*_*M*_ are shown in (**b**). (**c**) *ϕ*_*M*_ dependences of *μ*_0_*h*_z_ for [110] and [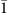
10] orientated devices. (**d**) Fitting coefficients of sin*ϕ*_*M*_ and cos*ϕ*_*M*_. The error bar in **b**,**d** is the s.d. obtained from the fit. All the fields are normalized by a unit current density of 10^11^ Am^−2^.

The presence of the component *V*_sym_ displayed in Fig. 2c implies a sizeable out-of-plane effective spin-orbit field (see equation (1)). Figure 2d shows the dependence of *V*_sym_ on *ϕ*_*M*_ for a current flowing along the [100] direction. The results cannot simply be fitted by sin2*ϕ*_*M*_ indicating that *h*^[001]^ depends on *ϕ*_*M*_. Figure 4a,c show the *ϕ*_*M*_ dependence of *h*^[001]^ for four crystallographic orientations. Here one can see that *h*^[001]^ strongly depends on *ϕ*_*M*_ and reaches a value as large as 0.35 mT for a current density of 10^11^ Am^−2^. We estimate that the magnitude of the SOT in our system is comparable to that observed in ferromagnetic metal/non-magnetic metal bi-layer systems^19^,^20^,^21^,^22^ (see Supplementary Note 9). Note that an additional contribution to the symmetric part of the dc voltage can in principle arise from spin pumping together with the spin galvanic effect. However, we calculate that this effect is two orders of magnitude smaller than the detected spin-orbit voltage.

To gain insight into the microscopic origin of the interfacial out-of-plane spin-orbit field, we show in Supplementary Note 8 that, within a two-dimensional spin-orbit ferromagnet model^32^, *h*^[001]^ is given by


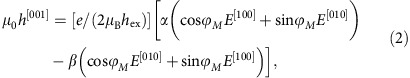


where *E*^[100]^, *E*^[010]^ is the electric field along the [100] and [010] directions, *e* the electronic charge, *μ*_β_ the Bohr magneton, and *h*_ex_ the exchange field. First, we estimate from this equation that the expected magnitude of *h*^[001]^ agrees well with the experiment (Supplementary Note 8). According to equation (2), the *ϕ*_*M*_ dependence of *h*^[001]^ can be explained by a combination of sin*ϕ*_*M*_ and cos*ϕ*_*M*_ terms depending on the symmetry of the SOI. The fitting coefficients of the sin*ϕ*_*M*_ and cos*ϕ*_*M*_ terms are shown in Fig. 4b,d (Supplementary Note 8). For the [100] and [010] directions, the cos*ϕ*_*M*_ and sin*ϕ*_*M*_ terms dominate, respectively. This indicates again that the Bychkov-Rashba SOI is the dominating SOI at the Fe/GaAs interface^13^. Moreover, the fitting coefficients for [110] and [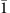
10] directions also agree well with the theoretical model of Bychkov-Rashba dominated SOI. From the fitting coefficients, the ratio of *α* and *β*, (*α*/*β*)_out-of-plane_, is determined. The value of (*α*/*β*)_out-of-plane_ for each device is larger than that of (*α*/*β*)_in-plane_ (see Supplementary Note 8). This is due to the different origins between in-plane and out-of-plane induced spin polarization. The in-plane spin polarization is created only at the Fermi level, while the out-of-plane spin polarization is due to the electrical polarization (intrinsic effect) of the whole bands^32^. Previous work on the ferromagnetic semiconductor (Ga,Mn)As^13^,^33^ explains an emerging *h*^[001]^ by the Kubo formalism, taking into account the presence of Dresselhaus dominated SOI and *p*-*d* exchange coupling. However, for our system of Fe/GaAs, *h*^[001]^ results from the broken symmetry at the interface and interfacial exchange coupling, a scenario which is distinctly different from the (Ga,Mn)As case.

### Measurement of the SGE

The effective spin-orbit fields stem from charge-to-spin conversion. In the following we demonstrate the inverse process, i.e., spin-to-charge conversion. In ferromagnetic metal/non-magnetic bilayers, spin pumping generates pure spin currents in the ferromagnetic layer. The pure spin current flows within the spin diffusion length into the non-magnetic layer and generates a transverse electrical current due to SOI, which is called ISHE^23^,^24^,^25^.

**Figure 5:**
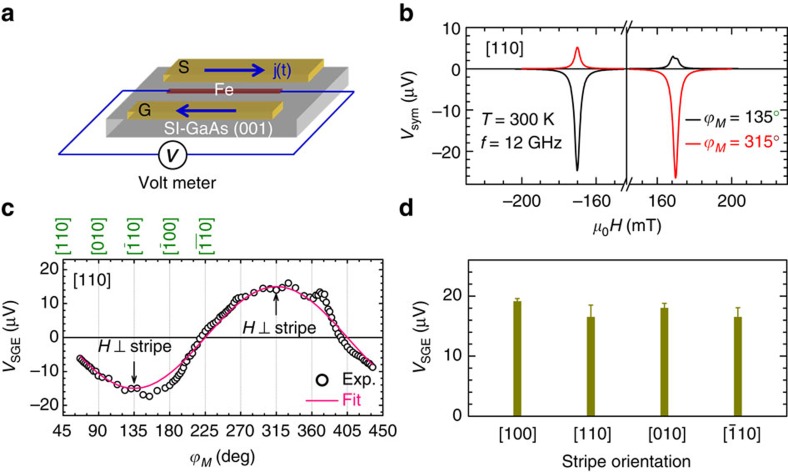
Spin-galvanic effect in Fe/GaAs. (**a**) Schematic of the device for spin pumping and spin-galvanic effect SGE. The [110]-orientated Fe/GaAs stripe is integrated between the signal (S) and ground (G) lines of the coplanar waveguide (CWG). (**b**) Magnetic-field *μ*_0_*H* dependent voltage measured at 300 K for *ϕ*_*M*_=135° and 315°. This is the ideal configuration for the study of SGE since the parasitic AMR of Fe is absent when *H* is perpendicular to the stripe. (**c**) Magnetization angle *ϕ*_*M*_ dependence of *V*_SGE_, the solid line is a fit to cos(*ϕ*_*M*_+45°). (**d**) Crystallographic orientation dependence of the magnitude of *V*_SGE_. The error bar is the s.d. obtained by fitting the *ϕ*_*M*_ dependence of *V*_SGE_ for different stripe orientations.

Figure 5a shows the schematic of the device used in our experiments. Fe/GaAs stripes with different orientations are integrated in the gap between the signal line and the ground plane of a coplanar waveguide. The stripe experiences an out-of-plane Oersted field excitation, which has been demonstrated to be an ideal configuration to study spin pumping and ISHE^34^,^35^. Figure 5b shows the measured voltage when the external magnetic field is in the plane but perpendicular to the stripe. For *ϕ*_*M*_=135°, the voltage has the opposite polarity at ±*H*_R_ but differs in magnitude, |*V*(−*H*_R_)|>|*V*(+*H*_R_)|. When the external magnetic field is rotated by 180°, the polarity reverses and |*V*(−*H*_R_)|<|*V*(+*H*_R_)| holds. This behaviour^36^ evidences the ISHE in ferromagnetic/non-magnetic layers and, in our case without the non-magnetic conducting layer, the presence of the SGE. The difference in the absolute magnitude at ±*H*_R_ indicates that a symmetric thermo-electric background^37^ coexists with the SGE, so that the magnitude of the spin-galvanic voltage *V*_SGE_ can be obtained by *V*_SGE_=[*V*(−*H*_R_)−*V*(*H*_R_)]/2 (see Supplementary Note 10). The *ϕ*_*M*_ dependence of *V*_SGE_, presented in Fig. 5c, can be fitted by cos(*ϕ*_*M*_+45°), concordant with the SGE^6^. Due to the *C*_*2v*_ symmetry of the system, the electric current flows always perpendicular to the spin polarization (magnetization)^6^. The angular dependence suggests that the Rashba SOI dominates the spin-to-charge conversion process. As shown in Fig. 5d, the SGE occurs in each orientations and the magnitude of *V*_SGE_ takes similar values for different orientations, indicating a similar strength of the Rashba SOI. An anisotropy in *V*_SGE_ could, in principle, be expected due to anisotropic scattering. However, according to the measurements of anisotropic tunnelling magnetoresistance^17^ and crystalline AMR^18^, the anisotropy is smaller than 1%. This should also be case for *V*_SGE_, which is beyond our detection limit.

## Discussion

We notice that in our system consisting of single crystalline Fe/GaAs, it is difficult to determine the magnitude of the spin current *J*_S_ injected into the interface by comparing the damping enhancement as commonly done for ferromagnetic/non-magnetic bilayers^38^,^39^ (for example, for Py/Pt bilayers, the damping constant is usually compared with that of Py/SiO_*x*_. The enhancement of damping is used to determine the spin mixing conductance and then *J*_S_). Recent experiments^26^ and microscopic theory^40^ suggest that the magnitude of *J*_S_ can be estimated by *J*_S_=*J*_C_/*λ*_SGE_, where *J*_C_=*I*_C_/*w* (*I*_C_ is the charge current density and *w* the width of the stripe) is an areal current density and *λ*_SGE_ the ‘effective thickness' of the spin-orbit layer. Our previous magneto-transport measurements^18^ show that the symmetry of AMR changes when the Fe layer reaches a characteristic thickness of ∼4 monolayers (0.57 nm). This is evidence for the presence of interfacial SOFs, which gives an upper limit of *λ*_SGE_. By using an average *J*_C_ of 6.7 × 10^−4^ Am^−1^, the lower bound of *J*_S_ is determined to be 1.2 × 10^6^ Am^−2^. This value is in good agreement with that in ferromagnetic/non-magnetic bilayers^26^,^34^,^41^ for a similar excitation. It should also be noted that at the Fe/GaAs interface both ferromagnetic exchange exists and SOI emerges. The generation of pure spin currents by spin pumping is due to the Fe exchange interaction and the spin-to-charge conversion occurs at the interfacial Fe layer due to SOI. The whole process occurs in one system. This is significantly different from any ferromagnet/non-magnetic bi-layer ever studied^25^,^26^,^27^,^30^,^34^,^35^,^36^,^37^,^38^,^39^,^41^, where the ferromagnetic and non-magnetic layer is separated and decoupled.

In summary, we have observed mutual conversion between charge and spin current, which is known as the SGE and inverse SGE, at an epitaxial Fe/GaAs interface by SO-FMR and spin pumping at room temperature. The magnitudes of the interfacial SOT in this single crystalline ferromagnetic metal/semiconductor system are found to be comparable to those at ferromagnetic metal metal/non-magnetic metal interfaces, which could be useful for future spintronic applications. Our findings open a pathway in search for efficient SOT in various single crystalline ferromagnetic metal/semiconductor interfaces.

## Methods

### Sample preparation

To guarantee high quality of the interfaces, Fe/GaAs samples are grown by MBE in a MBE cluster with *in-situ* transfer. First, an undoped GaAs buffer layer (100 nm) is deposited on top of a GaAs (001) semi-insulating substrate at 560 °C. Clear (2 × 4) surface construction pattern has been observed, indicating an As-terminated flat surface. Then, without breaking the vacuum, the sample is transferred to another MBE chamber, where a 5-nm Fe film is deposited on GaAs at a substrate temperature of 75 °C. Reflection high-energy electron diffraction oscillations have been observed during growth, indicating the epitaxial growth mode as well as the flat interface between Fe/GaAs. Finally, a 3-nm thick Al capping layer is deposited onto the Fe film to avoid oxidation. The high quality of the Fe films is evidenced by the small damping constant of 0.0036 (see Supplementary Note 3).

### Device

For the SO-FMR measurements, 6.4 × 100.0 μm stripes with different orientations are defined by electron-beam lithography and ion beam etching. The contacts are made from 15 nm Ti and 150 nm Au. All stripes have a resistance of ∼1.6 kΩ. For spin pumping measurements, 10.0 × 400.0 μm stripes with different orientations are integrated in the gap of the signal and ground lines of a coplanar wave guide. The resistances of the stripes are ∼2.6 kΩ. The width of the signal and ground line, which are also made from 15 nm Ti and 150 nm Au, is 50 and 30 μm, respectively.

### Measurements

For both SO-FMR and spin pumping measurements, microwave currents with a frequency of 12 GHz are used and the input microwave power is 22 dBm (∼158 mW). For SO-FMR measurements, a Bias Tee is used to separate the dc voltage from microwave background, and the driving power acting on the sample is estimated to be 65 mW. Since the skin depth at 12 GHz (∼145 nm) is much larger than the Fe thickness (5 nm), the current density in Fe is expected to be spatially uniform. Thus, the Oersted field should not produce torque on Fe itself. All measurements are performed at room temperature.

### Data availability

The data that support the findings of this study are available from the corresponding author on request.

## Additional information

**How to cite this article:** Chen, L. *et al*. Robust spin-orbit torque and spin-galvanic effect at the Fe/GaAs (001) interface at room temperature. *Nat. Commun.* **7,** 13802 doi: 10.1038/ncomms13802 (2016).

**Publisher's note:** Springer Nature remains neutral with regard to jurisdictional claims in published maps and institutional affiliations.

## Supplementary Material

Supplementary Information Supplementary Figures 1-12, Supplementary Tables 1-3, Supplementary Notes 1-10 and Supplementary References.
