# Supplementary material for: Robust spin-orbit torque and spin-galvanic effect at the Fe/GaAs (001) interface at room temperature
Source: Nat Commun. 2016 Dec 13;7:13802. doi: 10.1038/ncomms13802 (PMC5159805; doi:10.1038/ncomms13802)
Supplement: Supplementary Information — Supplementary Figures 1-12, Supplementary Tables 1-3, Supplementary Notes 1-10 and Supplementary References. [file ncomms13802-s1.pdf]

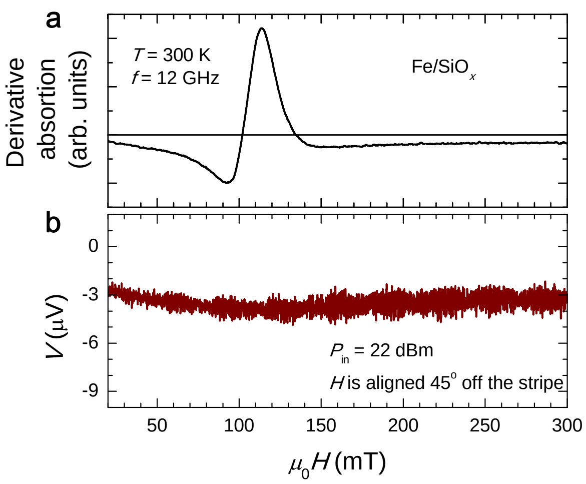

Supplementary Figure 1 | Ferromagnetic resonance spectrum and dc voltage for a polycrystalline Fe layer on SiO_x. (**a**) Ferromagnetic resonance spectrum of the bare Fe/SiO_x film. (**b**) The dc voltage spectrum of the SO-FMR device. No characteristic dc voltage is observed. The device has the same geometry as the Fe/GaAs devices. The external magnetic field is aligned ~45° off the stripe direction; in this configuration the magnitude of V is expected to be maximum.

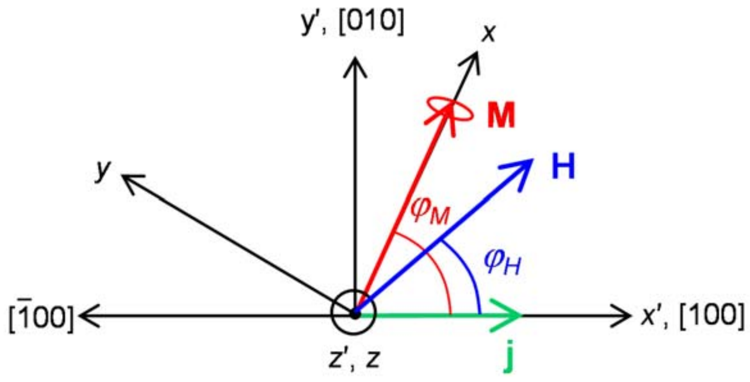

Supplementary Figure 2 | Cartesian coordinate systems used for the analysis. Two Cartesian coordinate systems (x', y', z') and (x, y, z) are used. As an example, the direction of the applied current and the detected voltage are along the [100]-direction. φ_M and φ_H are the angles of the magnetization and the magnetic field with respect to the [100]-direction.

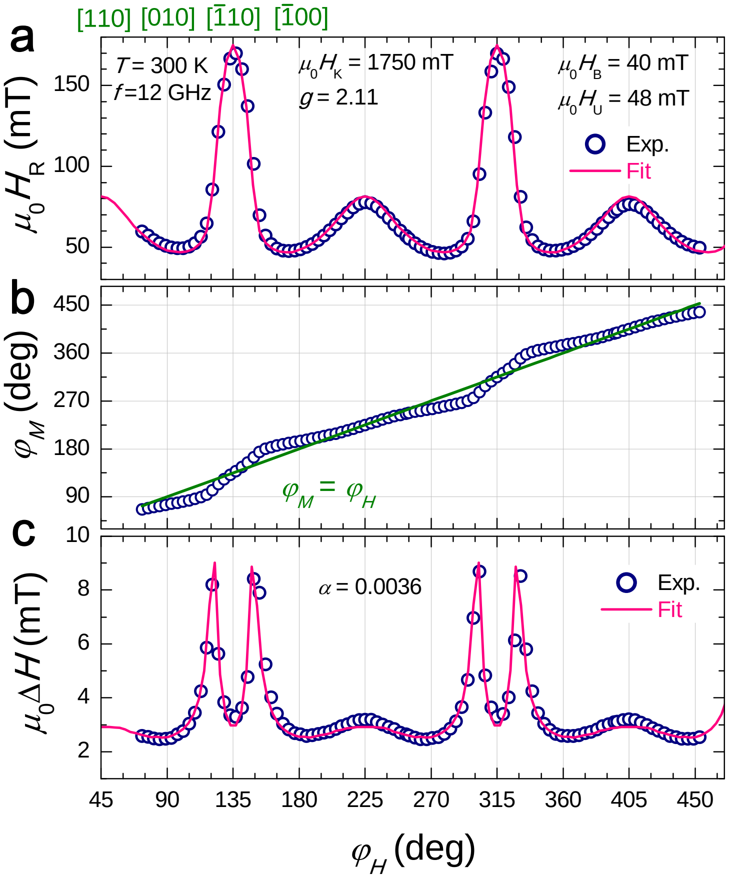

Supplementary Figure 3 | Magnetic-field angle dependence of the resonance field, magnetization angle and linewidth. (**a**) Magnetic-field angle φ_H dependence of the resonance field H_R. The solid line is a fit to Eq. 6. (**b**) φ_M as a function of φ_H calculated by Eq. 7 (open circles). The solid line represents the case of $\varphi_M = \varphi_H$. (**c**) Magnetic-field angle φ_H dependence of the linewidth ΔH. The solid line is calculated via Im(χ^1) (Eq. 9), and the resulting damping constant α is 0.0036.

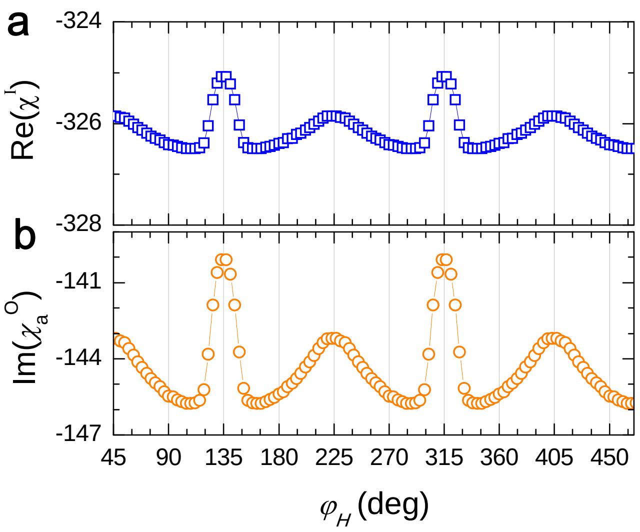

Supplementary Figure 4 | Calculated magnetic field angle dependence of susceptibility. (**a**) Re(χ^{I}) (**b**) Im(χ_a^{O}).

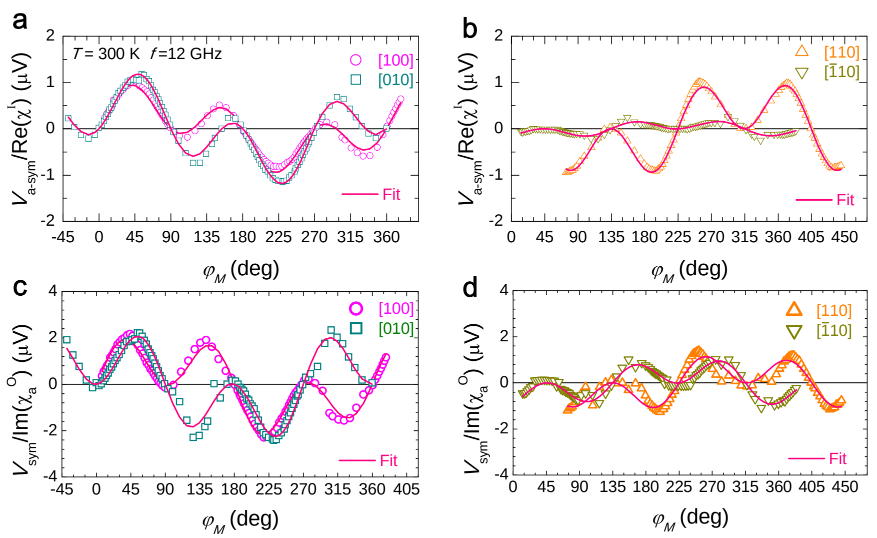

Supplementary Figure 5 | Magnetization angle dependence of the dc voltages. Magnetization angle φ_M dependence of $V_{\text{a-sym}}$ for [100] and [010] directions (**a**), and for [110] and [$\bar{1}$10] directions (**b**). The magnitude of the voltages is normalized by Re(χ^{I}). Magnetization angle φ_M dependence of V_{sym} for [100] and [010] directions (**c**), and for [110] and [$\bar{1}$10] directions (**d**). The magnitude of the voltage is normalized by Im($\chi_a{}^{\text{O}}$). The solid lines in are fits to Eqs. 2, 3 and 11.

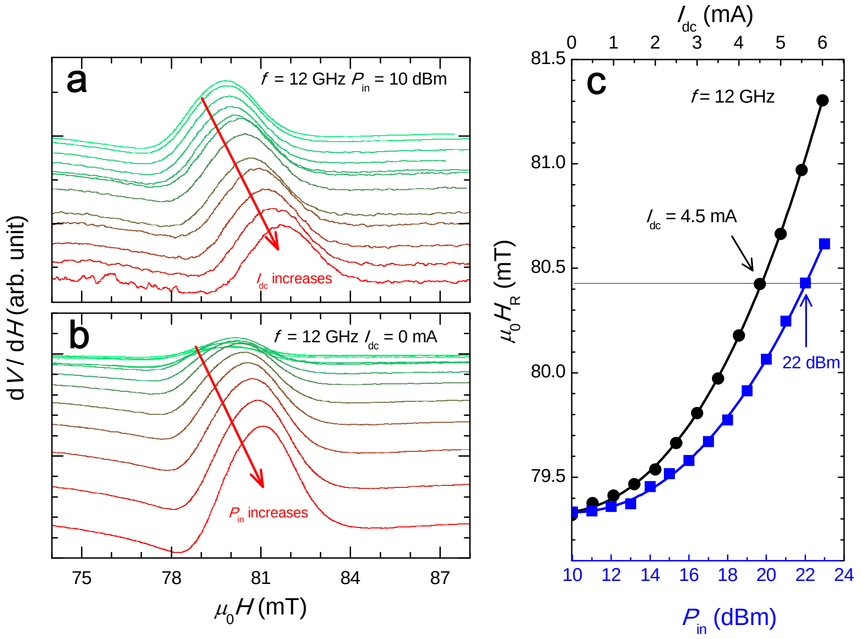

Supplementary Figure 6 | Calibration of the magnitude of the microwave current. (**a**) Derivative of the dc voltage obtained at microwave frequency f = 12 GHz, power P_{in} = 10 dBm as a function of the dc current I_{dc}. (**b**) Derivative of the dc voltage obtained at microwave frequency f = 12 GHz as a function of P_{in}. (**c**) Comparison between P_{in} dependence of H_R and I_{dc} dependence of H_R, from which the microwave current is determined.

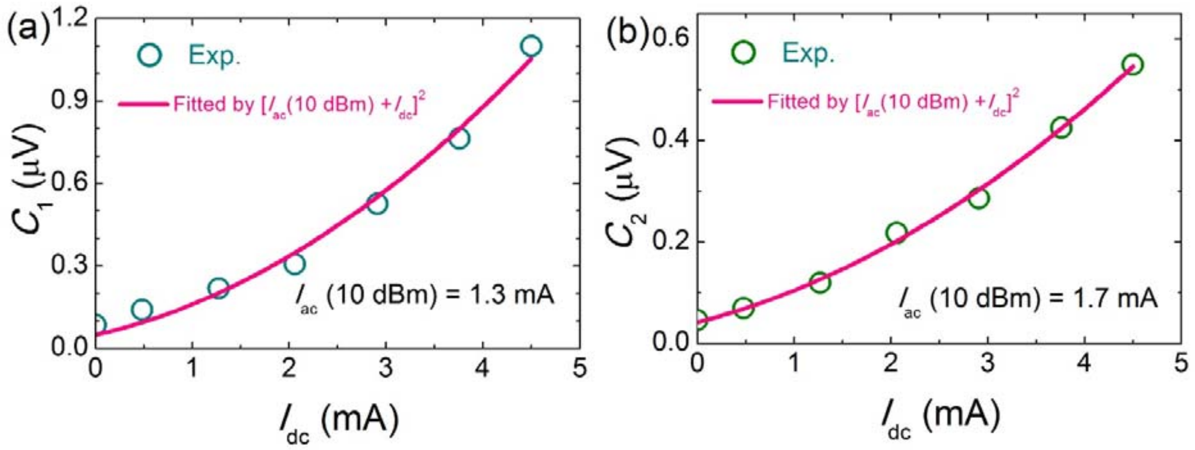

Supplementary Figure 7 | Determination of the microwave current at 10 dBm. I_{dc} dependence of C_1 (a) and C_2 (b). By fitting the experimental data by $k[I_{ac}(10\text{ dBm}) + I_{dc}]^2$, $I_{ac}(10\text{ dBm})$ is determined to be 1.3 mA for (a) and 1.7 mA for (b). We take an average value of 1.5 mA to calculate the total microwave current.

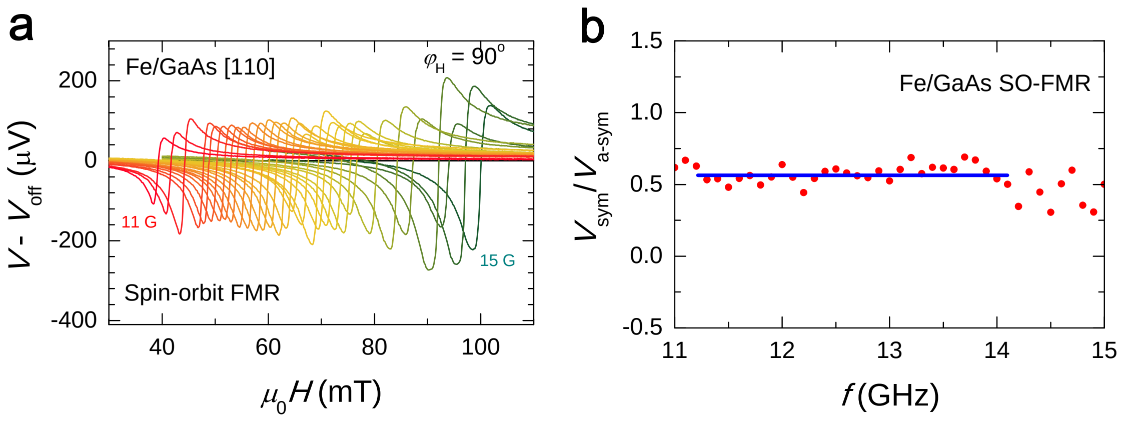

Supplementary Figure 8 | Frequency dependence of the lineshape of the dc voltage. (**a**) The dc voltage V spectrum as a function of microwave frequency measured from 11 GHz to 15 GHz. (**b**) Frequency dependence of $V_{sym}/V_{a\text{-}sym}$.

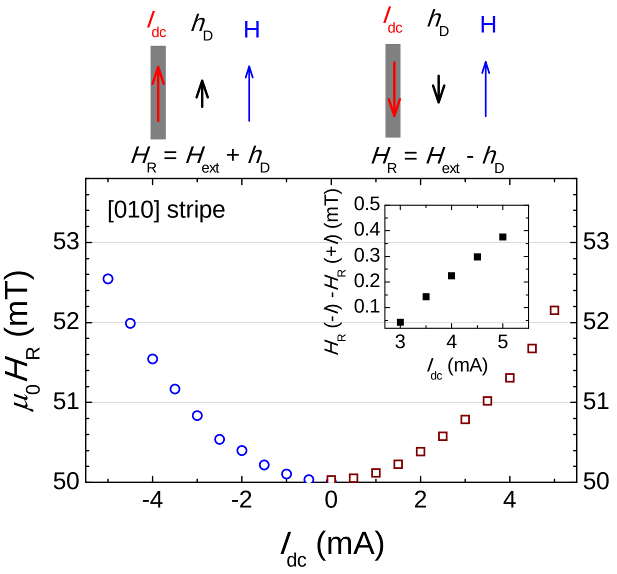

Supplementary Figure 9 | Further evidence for the existence of the Dresselhaus field. dc current I_{dc} dependence of the resonance field H_R for a [010]-orientated stripe. During measurement, the external field is along the stripe and the dc current is parallel ($-I_{dc}$) and anti-parallel ($+I_{dc}$) to the magnetic field. The inset shows the difference of H_R between $-I_{dc}$ and $+I_{dc}$, $H_R(-I_{dc}) - H_R(+I_{dc})$.

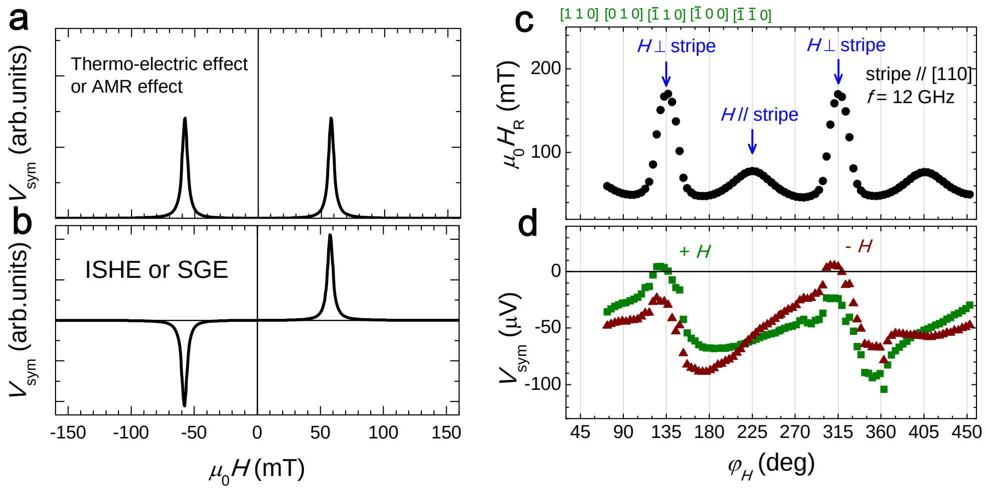

Supplementary Figure 10 | V_{sym} spectrum with respect to ±H symmetry. (**a**) Symmetric voltages originating from AMR or thermo-electric effects are symmetric with respect to H, $V_{sym}(-H) = V_{sym}(+H)$. (**b**) Symmetric voltages originating from ISHE or SGE are anti-symmetric with respect to the total magnetic-field, $V_{sym}(-H) = -V_{sym}(+H)$. (**c**) φ_H dependence of H_R used to show the direction of the external magnetic field with respect to the stripe. (**d**) φ_H dependence of V_{sym} (raw data) for Fe/GaAs. The stripe is oriented along the [110] direction.

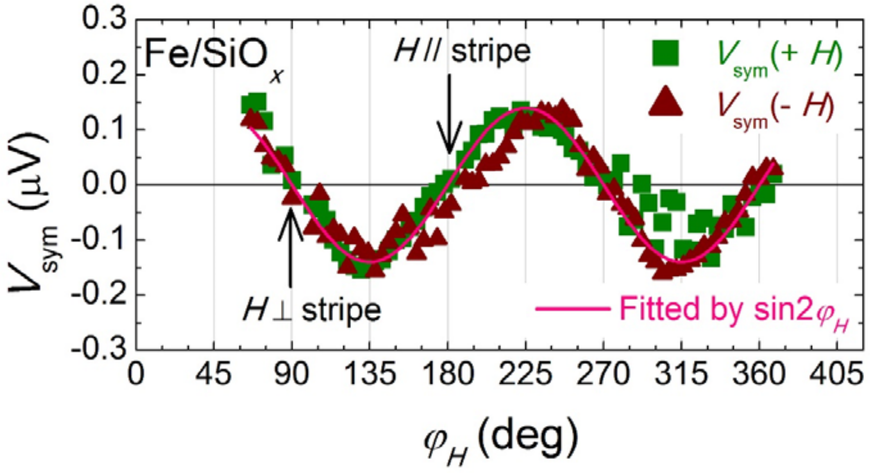

Supplementary Figure 11 | Magnetic-field angle dependence V_{sym} for Fe/SiO$_x$. The voltage goes to zero and no difference between $V_{sym}(+H)$ and $V_{sym}(-H)$ is observed when the magnetic field is perpendicular to the stripe, which indicates that no SGE exists for Fe/SiO$_x$. The solid line is a fit by the AMR effect.

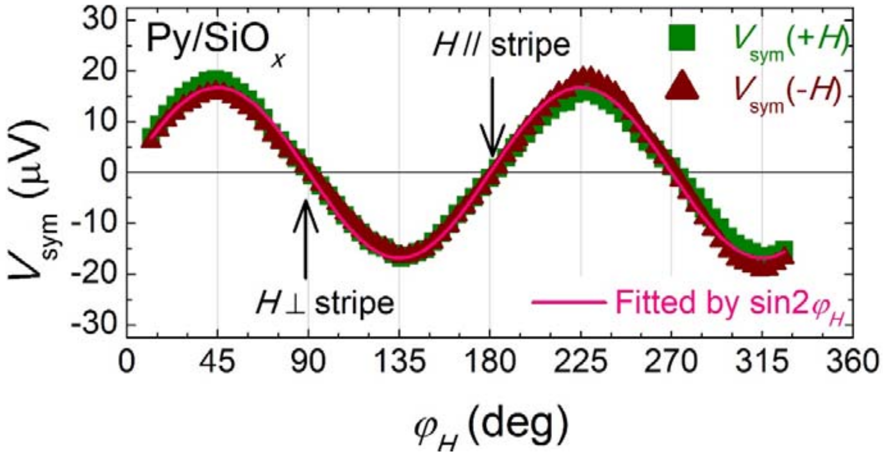

Supplementary Figure 12 | Magnetic-field angle dependence V_{sym} for Py/SiO$_x$. The voltage goes to zero and no difference between $V_{sym}(+H)$ and $V_{sym}(-H)$ is observed when the magnetic field is perpendicular to the stripe, which indicates that no SGE exists for Py/SiO$_x$. The solid line is a fit by the AMR effect.

Orientation	In-plane SOFs (mT)		Out-of-plane SOFs (mT)		j (10^{11} Am^{-2})
	$\mu_0 h^{[100]}$	$\mu_0 h^{[010]}$	sin compo.	cos compo.	
[100]	-0.15	0.28	-0.10	0.56	1.91
[010]	-0.27	0.14	0.70	-0.06	1.88
[110]	-0.17	0.43	0.40	0.52	2.13
[$\bar{1}$10]	-0.04	-0.03	0.46	-0.42	1.91

Supplementary Table 1 | Effective spin-orbit fields and microwave current density for devices oriented along different directions.

Orientation	$\mu_0 h^{[001]}$	sin	cos	$(\alpha/\beta)_{\text{out-of-plane}}$	$(\alpha/\beta)_{\text{in-plane}}$
[100]	$\frac{e}{4h_{ex}\mu_B}E^{100}(\alpha\cos\varphi_M - \beta\sin\varphi_M)$	–0.05	0.29	5.8	1.9
[010]	$\frac{e}{4h_{ex}\mu_B}E^{010}(\alpha\sin\varphi_M - \beta\cos\varphi_M)$	0.37	–0.03	12.7	2.0
[110]	$\frac{e}{4h_{ex}\mu_B}E^{110}\frac{\sqrt{2}}{2}[(\alpha-\beta)\cos\varphi_M + (\alpha-\beta)\sin\varphi_M]$	0.20	0.19	10.0	1.7
[$\underline{1}$10]	$\frac{e}{4h_{ex}\mu_B}E^{\bar{1}10}\frac{\sqrt{2}}{2}[-(\alpha+\beta)\cos\varphi_M + (\alpha+\beta)\sin\varphi_M]$	0.24	–0.22		

Supplementary Table 2 | Values of α/β for different stripe orientations. Fitting function of $\mu_0 h^{[001]}$ for different stripe orientations obtained from Eq. 2 in the manuscript. The ratio of α and β, α/β, is obtained from the fitting coefficient of sin and cos functions. α/β determined from in-plane SOFs (Fig. 3 in the main text) is also shown.

**FM/NM**	t_{FM} **(nm)**	$\mu_0 h$ **(mT)**	j **(10^{11} Am^{-2})**	$\mu_0 h * t_{FM}/j$ **(mT.nm/10^{11} Am^{-2})**	Ref.
CoFeB/Pt	0.5-5.7	0.6-0.06	0.2	1.5	14
Co/Pt	0.6	5	1	3.0	15
CoFeB/Ta	0.9	3	1	2.7	15,16
CoNi/PtMn	2.1	7.8	1	16.4	17
Fe/(Ga,Mn)As	2	0.05	0.1	1	18
Fe/GaAs	5	0.35	1	1.8	Our results

Supplementary Table 3 | Comparison of various ferromagnetic metal/non-magnetic bi-layers.

SUPPLEMENTARY NOTES

Supplementary Note 1: Estimation of the influence of the current induced Oersted field

To demonstrate that the observed dc voltage is indeed induced by the effective spin-orbit fields (eSOFs) at the interface of single crystalline Fe/GaAs, we measure a polycrystalline 5.6-nm thick Fe film deposited on an amorphous SiO_x substrate. The ferromagnetic resonance (FMR) absorption of the bare Fe/SiO_x film, measured at 12 GHz, is presented in Supplementary Fig. 1a. One can see that a well-defined FMR line appears at a resonance field of H_R ~ 110 mT. However, no characteristic dc voltage V is observed for the spin-orbit FMR (SO-FMR) device with the same device dimensions and the same microwave excitation as the single crystalline Fe/GaAs devices (Supplementary Fig. 1b). This indicates that the observed dc voltages cannot arise from the current induced Oersted field, but stem from the eSOFs at the single crystalline Fe/GaAs interface.

Supplementary Note 2: dc voltages along different crystallographic orientations

As shown in Supplementary Fig. 2, we use two coordinate systems to derive the angular dependence of the dc voltages. In the measurement coordinate system (x', y', z'), the microwave current density **j** flows along the [100]-orientation and the dc voltage is also detected along this direction. In the coordinate system labelled (x, y, z), the magnetization **M** and the microwave current **j** can be described as $\mathbf{M} = (M_x, m_y e^{i\omega t}, m_z e^{i\omega t})$, and $\mathbf{j} = j e^{i\omega_0 t}(\cos\varphi_M, -\sin\varphi_M, 0)$, where M_x is the static magnetization along the x-direction, m_y (m_z) the dynamic magnetization along the y (z)-direction, ω the angular frequency of the magnetization precession, ω_0 the angular frequency of the driving current, j the magnitude of the microwave current density, and φ_M the magnetization angle. The phenomenological relationship between the electric field **E** and **j** can be expressed as[1]

$$\mathbf{E} = \rho\mathbf{j} + \Delta\rho\mathbf{n}(\mathbf{n}\cdot\mathbf{j}) + \rho_{\mathrm{H}}\mathbf{n}\times\mathbf{j}, \qquad (1)$$

where ρ is the resistivity, $\Delta\rho$ magnitude of the anisotropic magneto-resistance AMR, $\mathbf{n}$ ($\mathbf{n} = \mathbf{M}/M$) the unit vector of the dynamic magnetization, and ρ_H the anomalous Hall resistivity. The first term on the right-hand side of Eq. 1 corresponds to Ohm's law, the second term to the AMR effect, and the third term to the anomalous Hall effect. Note that the crystalline AMR effect[2], which only contributes significantly when the Fe thickness is reduced to a few monolayers, is not taken into account here because of the thick Fe film used in this study (~35 monolayers). For SO-FMR measurements, only the AMR effect is taken into account since the direction of the detected dc voltage is along the current direction. For the [100] orientated device, the dc voltage V can be derived from Eq. 1,

$$V^{[100]} = \Delta\rho l\overline{[\mathbf{n}(\mathbf{n}\cdot\mathbf{j})]_{x'}} = -\frac{\Delta\rho jl}{2M}\mathrm{Re}(m_y)\sin 2\varphi_M, \qquad (2)$$

where the overline denotes the time average, $\mathrm{Re}(m_y)$ the real part of the dynamic magnetization m_y, and l the length of the device. Similarly, the voltage V for other directions can be obtained as,

$$\begin{aligned} V^{[010]} &= \frac{\Delta\rho jl}{2M}\mathrm{Re}(m_y)\sin 2\varphi_M, \\ V^{[110]} &= \frac{\Delta\rho jl}{2M}\mathrm{Re}(m_y)\cos 2\varphi_M, \\ V^{[\bar{1}10]} &= -\frac{\Delta\rho jl}{2M}\mathrm{Re}(m_y)\cos 2\varphi_M. \end{aligned} \qquad (3)$$

Supplementary Note 3: Ferromagnetic resonance results and dynamic magnetic susceptibility

To obtain the magnitude and lineshape of $\mathrm{Re}(m_y)$, we solve the Landau-Lifshitz-Gilbert (LLG) equation analytically, which has been widely used to describe magnetization dynamics[3]:

$$\frac{\mathrm{d}\mathbf{M}}{\mathrm{d}t} = -\mu_0\gamma\mathbf{M}\times\mathbf{H}_{\text{eff}} + \frac{\alpha}{|\mathbf{M}|}\mathbf{M}\times\frac{\mathrm{d}\mathbf{M}}{\mathrm{d}t}, \tag{4}$$

where μ_0 is the permeability in vacuum, γ the gyromagnetic ratio, α the damping constant, $\mathbf{H}_{\text{eff}}$ the effective magnetic field, which is the sum of the external magnetic field H, anisotropy fields, their dynamic components, as well as current induced effective spin-orbit fields. The first term on the right-hand side of Eq. 4 describes the precession of **M**, and the second term is the damping term which describes the relaxation of **M**. In the coordinate system (x, y, z), $\mathbf{H}_{\text{eff}}$ can be derived as

$$\begin{aligned} H_{eff}^{x} &= H\cos(\varphi_M - \varphi_H) + H_B\cos 4\varphi_M - H_U\sin 2\varphi_M + \left(h^{[100]}\cos\varphi_M + h^{[010]}\sin\varphi_M\right)e^{i\omega_0 t}, \\ H_{eff}^{y} &= \left(-h^{[100]}\sin\varphi_M + h^{[010]}\cos\varphi_M\right)e^{i\omega_0 t}, \\ H_{eff}^{z} &= \left[-\frac{3}{2}H_B\sin^2 2\varphi_M - H_U\cos^2\left(\varphi_M - \frac{\pi}{4}\right) - H_K\right]\frac{m_z e^{i\omega t}}{M_x} + h^{[001]}e^{i\omega_0 t}, \end{aligned} \tag{5}$$

where $h^{[100]}$, $h^{[010]}$, $h^{[001]}$ are eSOFs along the [100], [010] and [001] orientations, H_B the biaxial in-plane magnetic anisotropy field along ⟨100⟩, H_U the uniaxial in-plane magnetic field along [110], and H_K the effective perpendicular uniaxial magnetic anisotropy field including the demagnetization field along [001].

By solving Eq. 4 without damping term, one can obtain the resonance condition[4]

$$\left(\frac{\omega}{\gamma}\right)^2 = \mu_0^2 H_1 H_2, \tag{6}$$

with $H_1 = H\cos(\varphi_M - \varphi_H) + H_K + H_B(3 + \cos 4\varphi_M)/4 - H_U\cos 2\varphi_M$, and $H_2 = H\cos(\varphi_M - \varphi_H) + H_B\cos 4\varphi_M - H_U\sin 2\varphi_M$. Supplementary Figure 3a shows the magnetic-field angle φ_H dependence of the resonance field H_R obtained at f = 12 GHz. The results indicate strong in-plane anisotropy with the easiest axis along ⟨100⟩ and the hardest axis along $[\bar{1}10]$, typical for thin Fe layers grown on GaAs (001). The results can be well reproduced by Eq. 6 using the fitting parameters of $\mu_0 H_K$ = 1750 mT, $\mu_0 H_B$ = 48 mT, $\mu_0 H_U$ = 45 mT, and g = 2.11, which are reasonable values for Fe layers on GaAs (001) in this thickness range[4]. Supplementary Figure 3b shows the magnetization angle φ_M as a function of magnetic-field angle φ_H, which is obtained from the equilibrium condition

$$H_{\mathrm{R}}\sin(\varphi_M - \varphi_H) + (H_{\mathrm{B}}/4)\sin 4\varphi_M + (H_{\mathrm{U}}/2)\cos 2\varphi_M = 0. \tag{7}$$

One can see that the magnetization direction φ_M deviates from the magnetic-field direction φ_H for all angles except for those along the [110] and [$\bar{1}$10] directions, and the magnitude of the deviation is larger around the hard axis.

If we solve Eq. 4, by assuming $\alpha \ll 1$, $M_{\mathrm{x}} = |\mathbf{M}|$ and ignore second order terms[3], one can obtain the complex dynamic magnetic susceptibility χ, which is related to the dynamic magnetization **M** and the eSOFs $\mathbf{h_{so}}$

$$\begin{pmatrix} m_y \\ m_z \end{pmatrix} = \begin{pmatrix} \chi^{\mathrm{I}} & -\mathrm{i}\chi_{\mathrm{a}}^{\mathrm{O}} \\ \mathrm{i}\chi_{\mathrm{a}}^{\mathrm{I}} & \chi^{\mathrm{O}} \end{pmatrix} \begin{pmatrix} -h^{[100]}\sin\varphi_M + h^{[010]}\cos\varphi_M \\ h^{[001]} \end{pmatrix}, \tag{8}$$

where χ^{I} ($\chi_{\mathrm{a}}^{\mathrm{I}}$) is the complex diagonal (off-diagonal) dynamic magnetic susceptibility due to the in-plane excitation by $h^{[100]}$ and $h^{[010]}$, χ^{O} ($\chi_{\mathrm{a}}^{\mathrm{O}}$) the complex diagonal (off-diagonal) dynamic magnetic susceptibility due to the out-of-plane excitation $h^{[001]}$. Each component of the susceptibility χ has both real and imaginary parts, $\chi = \mathrm{Re}(\chi) + \mathrm{iIm}(\chi)$, which can be written as

$$\begin{aligned}
\mathrm{Re}\left(\chi^{\mathrm{I}}\right) &= -\frac{H_1(H_1^R H_2^R - H_1 H_2)M_x}{(H_1 H_2 - H_1^{\mathrm{R}} H_2^{\mathrm{R}})^2 + \alpha^2 H_1^R H_2^R (H_1 + H_2)^2}, \\
\mathrm{Im}\left(\chi^{\mathrm{I}}\right) &= \frac{\alpha\sqrt{H_1^R H_2^R}[H_1 H_1 + H_1^R H_2^R]M_x}{(H_1 H_2 - H_1^{\mathrm{R}} H_2^{\mathrm{R}})^2 + \alpha^2 H_1^R H_2^R (H_1 + H_2)^2}, \\
\mathrm{Re}\left(\chi_{\mathrm{a}}^{\mathrm{I}}\right) &= \frac{\sqrt{H_1^R H_2^R}(H_1^R H_2^R - H_1 H_2)M_x}{(H_1 H_2 - H_1^{\mathrm{R}} H_2^{\mathrm{R}})^2 + \alpha^2 H_1^R H_2^R (H_1 + H_2)^2}, \\
\mathrm{Im}\left(\chi_{\mathrm{a}}^{\mathrm{I}}\right) &= -\frac{\alpha H_1^R H_2^R (H_1 + H_2)M_x}{(H_1 H_2 - H_1^{\mathrm{R}} H_2^{\mathrm{R}})^2 + \alpha^2 H_1^R H_2^R (H_1 + H_2)^2},
\end{aligned} \tag{9}$$

and

$$
\begin{aligned}
\mathrm{Re}\left(\chi^{\mathrm{O}}\right) &= -\frac{H_2(H_1^R H_2^R - H_1 H_2)M_x}{(H_1 H_2 - H_1^{\mathrm{R}} H_2^{\mathrm{R}})^2 + \alpha^2 H_1^R H_2^R (H_1 + H_2)^2}, \\
\mathrm{Im}\left(\chi^{\mathrm{O}}\right) &= \frac{\alpha\sqrt{H_1^R H_2^R}[H_2 H_2 + H_1^R H_2^R]M_x}{(H_1 H_2 - H_1^{\mathrm{R}} H_2^{\mathrm{R}})^2 + \alpha^2 H_1^R H_2^R (H_1 + H_2)^2}, \\
\mathrm{Re}\left(\chi_{\mathrm{a}}^{\mathrm{O}}\right) &= \frac{\sqrt{H_1^R H_2^R}(H_1^R H_2^R - H_1 H_2)M_x}{(H_1 H_2 - H_1^{\mathrm{R}} H_2^{\mathrm{R}})^2 + \alpha^2 H_1^R H_2^R (H_1 + H_2)^2}, \\
\mathrm{Im}\left(\chi_{\mathrm{a}}^{\mathrm{O}}\right) &= -\frac{\alpha H_1^R H_2^R (H_1 + H_2)M_x}{(H_1 H_2 - H_1^{\mathrm{R}} H_2^{\mathrm{R}})^2 + \alpha^2 H_1^R H_2^R (H_1 + H_2)^2},
\end{aligned}
\qquad (10)
$$

where H_1^R and H_2^R are H_1 and H_2 at resonance H_R. Note the slight difference between diagonal components χ^I and χ^O. Since the FMR absorption is determined by Im(χ), the magnetic-field angle φ_H dependence of $\mu_0\Delta H$ can be well reproduced by calculating the linewidth of Im(χ) by adopting α as a fitting parameter[3] (Supplementary Fig. 3c). In the calculation, the φ_H dependence of $\mu_0 H_R$ and φ_M is used. The obtained value of α ~ 0.0036, is smaller than for most metallic ferromagnets. This low damping is an indication of the high quality of our samples. The reasonable fit as well as the small linewidth (~2.5 mT for the ⟨100⟩ orientations) make us believe that the main contribution to the linewidth is intrinsic, and the extrinsic contributions[4-6], such as inhomogeneous broadening and/or two-magnon scattering, are small for the present sample.

From Eqs. 8-10, the real part of m_y, Re(m_y), can be obtained as

$$
\mathrm{Re}(m_y) = \mathrm{Re}(\chi^I)(-h^{[100]}\sin\varphi_M + h^{[010]}\cos\varphi_M) - \mathrm{Im}(\chi_a^O)h^{[001]}. \qquad (11)
$$

It is known that Re(χ^I) has an anti-symmetric lineshape while Im(χ_a^O) has a symmetric lineshape. Thus, from Equations 2, 3 and 11 we realize that the anti-symmetric part of the dc voltage $V_{a\text{-}sym}$ is only related to the in-plane effective spin-orbit fields ($h^{[100]}$ and $h^{[010]}$), while the symmetric part of the dc voltage V_{sym} is only related to the out-of-plane spin-orbit field ($h^{[001]}$). The magnitudes of Re(χ^I) and Im(χ_a^O) calculated from Eqs. 9 and 10 are presented in Supplementary Fig. 4. One can see that the absolute value of Re(χ^I) is about 2 times larger than Im(χ_a^O). This is expected since the magnetic easy axis is in the plane. The magnitude of the susceptibility is also found to be angle dependent, which results from the in-plane magnetic anisotropy as well as the angular variation of the linewidth (Supplementary Figs. 3a and c).

Supplementary Note 4: Magnetization angle dependence of the dc voltages

Supplementary Figure 5 shows the magnetization angle φ_M dependence of the dc voltages for stripes prepared along different orientations. Since the magnitude of Re(χ^I) and Im(χ_a^O) is angle dependent (Supplementary Fig. 4), $V_{a\text{-sym}}$ is normalized by Re(χ^I) and V_{sym} is normalized by Im(χ_a^O). The results can be well fitted by combining Eqs. 2, 3 and 11, and the resulting eSOFs are listed in Supplementary Table 1. It is noted in Supplementary Figure 5b that the magnitude of $V_{a\text{-sym}}$ along the [$\bar{1}$10] direction is about 5 times smaller than that along the [110] direction, since Dresselhaus and Bychkov-Rashba fields have opposite directions for [$\bar{1}$10], but the same direction for [110]. This large difference in amplitude also excludes the possibility that the Oersted field is the main contributor to the driving field.

Supplementary Note 5: Calibration of the magnitude of the microwave current

We use the resonance field shift induced by Joule heating to determine the magnitude of the microwave current. First, we measure the voltage spectrum at low microwave power by introducing a dc current I_{dc} through the Bias Tee. The application of I_{dc} results in a large background signal of ~ 1 V. To increase the sensitivity, we modulate the magnetic field by ~ 1 mT at a frequency of 86 Hz, and measure dV/dH using a lock-in amplifier. Supplementary Figure 6a shows the dV/dH curves as a function of I_{dc} measured at P_{in} = 10 dBm. It can be seen that H_R shifts to higher field values as I_{dc} increases. A similar behaviour is observed when increasing the microwave power (Supplementary Figure 6b). The dependence of H_R on P_{in} and I_{dc} is summarized in Supplementary Figure 6c. The quadratic dependence indicates that the origin of the shift of H_R is due to Joule heating. By comparing these two quantities, we obtain the microwave current I_{dc} at each P_{in}. It should be noted that for this method the total current still contains a small ac current I_{ac} due to the applied low microwave power (10 dBm). We determine I_{ac} (10 dBm) in the following way: First, we measure

the angular dependence of the dc voltage for each P_{in}. The anti-symmetric part of the dc voltage can be fitted by $V_{a-sym}^{[010]} / \mathrm{Re}(\chi^{I}) = C_1 \cos\varphi_M \sin 2\varphi_M + C_2 \sin\varphi_M \sin 2\varphi_M$, where C_1 and C_2 is proportional to j and h_{SO} (according to Eqs. 3 and 11). From the fitting, C_1 as a function of P_{in} is obtained. Second, due to the $P_{in} \sim I_{dc}$ calibration shown in Supplementary Figure 6c, the $C_1 \sim P_{in}$ relationship can be transferred to a $C_1 \sim I_{dc}$ relationship (Supplementary Fig. 7). Third, the total current I_{tot} is sum of I_{ac}(10 dBm) and I_{dc}, $I_{tot} = I_{ac}$(10 dBm) + I_{dc}. Fourth, because the magnitude of the dc voltage (~C_1) is proportional to $I_{tot}h_{SO}$ (see Eq. 1 in the main text) and the magnitude of the spin-orbit field h_{SO} is proportional to I_{tot}, which can be expressed as $C_1/I_{tot} \sim h_{SO} \sim I_{tot}$, and we have $C_1 \sim I_{tot}^2 \sim [I_{ac}(10\ \mathrm{dBm}) + I_{dc}]^2$. Fifth, as shown in Supplementary Figure 7, I_{dc} dependence of C_1 and C_2 is fitted by $[I_{ac}(10\ \mathrm{dBm}) + I_{dc}]^2$, and I_{ac} is determined to be 1.3 mA and 1.7 mA, respectively. Finally, taking an average value of 1.5 mA, we obtain a total microwave current, $I_{tot} = I_{ac}$(10 dBm) + I_{dc} = 1.5mA + 4.5mA = 6 mA. The current densities for all the devices are listed in Supplementary Table 1.

Supplementary Note 6: Frequency dependence of the lineshape of the dc voltage

Supplementary Figure 8a shows $V(H)$ spectra measured as a function of frequency. One can see that the magnitude of the dc voltage varies only slightly for a wide range of frequencies between 11GHz and 15GHz except for the smallest frequencies around 11GHz and the largest frequencies above 14GHz. The amplitude variation may arise from the fact that the transmission of microwave power through cables and/or bonding contacts is frequency dependent. The ratio of the symmetric to anti-symmetric components, V_{sym}/V_{aym}, is used to describe the line shape. As shown in Supplementary Figure 8b, one can see that the magnitude of V_{sym}/V_{aym} remains constant for a wide range of frequencies, indicating that the phase difference between **j** and **h** is negligibly small due to the fact that **j** and **h** are intrinsically coupled for SO-FMR. This behaviour can be compared to the line shape of the dc voltages measured by Oersted field driven FMR in the CPW (similar to Fig. 5a in the main text), where the magnitude and sign of V_{sym}/V_{aym} vary strongly due to the frequency induced phase shift[7].

Supplementary Note 7: Further evidence for the existence of the Dresselhaus field

Here we show that the direction and magnitude of the Dresselhaus spin-orbit field can be independently verified by the shift of the resonance field H_R in a [010] (or [100]) orientated device. To do so, we set the external magnetic field along the stripe direction, and measure the dV/dH spectrum by tuning the magnitude and direction of the dc current I_{dc}. Two points should be noted for this configuration: i) the dc voltage is still sizeable because of the deviation between magnetization and magnetic-field angle (φ_H = 90° but φ_M = 76° as shown in Supplementary Fig. 3b). ii) Only the Dresselhaus field can be detected since the external field points along the stripe. Supplementary Figure 9 shows the dc current I_{dc} dependence of resonance field H_R. We observe that the magnitude of $H_R(-I_{dc})$ is slightly larger than that of $H_R(+I_{dc})$. This asymmetric behaviour is expected when the Dresselhaus field is parallel to H for $-I_{dc}$, and anti-parallel to H for $+I_{dc}$. The inset shows the I_{dc} dependence of the difference of the measured resonance fields $H_R(-I_{dc}) - H_R(+I_{dc})$. As can be seen, the relation is linear for currents larger than 3 mA. The magnitude of the Dresselhaus field, $[H_R(-I_{dc}) - H_R(+I_{dc})]/2$, is determined to be 0.16 mT for I_{dc} = 5 mA (corresponding to $j = 1.6\times10^{11}$ Am^{-2}), which is in good agreement with the value determined by SO-FMR (Supplementary Table 1 and Fig. 3b in the main text).

Supplementary Note 8: Theoretical estimation of the out-of-plane effective spin-orbit fields

The out-of-plane component of the spin accumulation $s^{[001]}$ can arise from virtual transitions between the exchange-split bands, induced by the electric field **E** established in the sample by the current flow. The virtual transitions are due to the spin-orbit velocity coming from the

spin-orbit fields[8-10]. In the presence of disorder, $s^{[001]}$ acquires a contribution from scattering[11], but the magnitude for the realistic case changes only little. For a generic spin-orbit field $\mathbf{w_k}$ with in-plane exchange interaction $\mathbf{h}_{ex}$, the total Hamiltonian can be written as $H = \varepsilon_k + \mathbf{h}_{ex}\cdot\boldsymbol{\sigma} + \boldsymbol{\sigma}\cdot\mathbf{w_k}$, here ε_k is the kinetic energy, $\mathbf{h}_{ex} = h_{ex}(m_x, m_y)$, with $m_x = \cos\varphi_M$ and $m_y = \sin\varphi_M$. $s^{[001]}$ can be calculated from the perturbation theory to be

$$s^{[001]} \approx \frac{eE_i\hbar g_F}{4h_{ex}} \left\langle m_x \nabla_{ki} w_{ky} - m_y \nabla_{ki} w_{kx} \right\rangle_{FS}, \tag{12}$$

where i represents x', y', and the average is over the Fermi surface (FS), and g_F is the density of states per unit volume at the Fermi level ε_F. In the formula we assume $w_k << h_{ex} << \varepsilon_F$ which is certainly valid for Fe/GaAs slabs. Higher-order terms in w_k/h_{ex} lead to higher harmonics in $s^{[001]}$, which become more prominent for materials with small exchange, such as the ferromagnetic semiconductor (Ga,Mn)As. In the simplified model of linear (in momentum k) Rashba and Dresselhaus coupling, $\mathbf{w_k} = [(-\alpha + \beta)(\sqrt{2}/2)(-k_{x'} + k_{y'}), (\alpha + \beta)(\sqrt{2}/2)(k_{x'} + k_{y'}),]$, we get $s^{[001]} = (e\hbar g_F/4h_{ex})\ [\alpha(\cos\varphi_M E_{x'} + \sin\varphi_M E_{y'}) + \beta(\cos\varphi_M E_{y'} + \sin\varphi_M E_{x'})]$. A similar expression for the Rashba-only case was obtained in Ref. 11, and for non-equilibrium in-plane spins, including both Rashba and Dresselhaus terms, in Ref. 12. The effective spin-orbit field $h^{[001]}$ corresponding to the effective spin accumulation is then given by $\mu_0 h^{[001]} = 2s^{[001]}/\hbar\mu_B g_F$.

From the observed magnitude of the induced field we get the Fermi-surface magnitude of the spin-orbit field per Fermi wave vector $w_k/k_F \approx (h^{[001]}\hbar h_{ex}/m_e E)$, where m_e is the electron mass. Taking $h_{ex} \approx 1$ eV for Fe, and $E \approx 10^4$ V/m, we get $w_k/k_F \approx 100$ meV Å for the measured ~1 mT field, which is in the range of values of the spin-orbit fields in Fe-GaAs obtained from first principles[13].

The φ_M dependence of $h^{[001]}$ also agrees well with our measurements as shown in Figs. 4a and

c in the manuscript. Supplementary Table 2 shows the fitting function of $\mu_0 h^{[001]}$ for different stripe orientations, which is a combination of sin and cos functions. The fitting coefficients of sin and cos are listed in the table from which the ratio of α and β, $(\alpha/\beta)_{\text{out-of-plane}}$, is determined. For comparison, values of α/β determined from in-plane eSOFs (Figs. 3a and b in the main text), $(\alpha/\beta)_{\text{in-plane}}$, are also presented. One can see that $(\alpha/\beta)_{\text{out-of-plane}}$ and $(\alpha/\beta)_{\text{in-plane}}$ are of the same sign and that the value of both is larger than 1 for all the devices, which indicates that the Rashba SOI dominates at the Fe/GaAs interface. The value of $(\alpha/\beta)_{\text{out-of-plane}}$ is larger than that of $(\alpha/\beta)_{\text{in-plane}}$. This discrepancy is due to the different origins between in-plane and out-of-plane induced spin polarization. The in-plane spin polarization is created only at the Fermi level, while the out-of-plane spin polarization is due to the electrical polarization (intrinsic effect) of the whole bands[11].

Supplementary Note 9: Magnitude of spin-orbit torque in ferromagnetic metal/non-magnetic metal bi-layers

The spin-orbit torque (SOT) is defined by $\mu_0\mathbf{h}_{\text{SO}}\times\boldsymbol{\mu}$, where $\boldsymbol{\mu}$ is the magnetic moment which increases with film thickness t_{FM} while $|\mathbf{h}_{\text{SO}}|$ decreases[14]. To compare with previous experiments we assume the magnetization of all the different ferromagnets to be the same and consider only the thickness of the ferromagnetic films. Furthermore, we normalize the spin-orbit field by the current density j. The resulting values of this rough estimate are listed in Supplementary Table 3.

Supplementary Note 10: Determination of the magnitude of the dc voltage induced by spin pumping

In ferromagnetic/non-magnetic bilayers, a long-standing issue has been the accurate determination of the magnitude of the voltage V_{SP} induced by spin pumping. The difficulty lies in excluding parasitic effects, e.g., spin rectification effects (Refs. 4 and 19, and references therein),

thermo-magnetic effects in the ferromagnetic layers[20] and the effect of spin memory loss at the ferromagnetic/nonmagnetic interface[21]. For the device we use here with the stripe integrated between the signal line and ground line (Fig. 5a in the main text), it has already been well established by using Py/Pt that V_{SP} and rectification voltages show different angular dependencies. Especially, V_{SP} has a maximum value when the external in-plane magnetic field H is perpendicular to the stripe while rectification voltage vanishes for this geometry[22,23].

Since spin pumping generates a pure spin current with symmetric line shape with respect to the external magnetic field H, usually, only V_{sym} is taken into account in the analysis. It has been shown, however, that there are three possible origins for V_{sym}: Rectification effects in the ferromagnetic layer due to AMR[19], thermo-magnetic effects in the ferromagnetic layer[20], as well as V_{SP}. Supplementary Figures 10a and b show the magnetic field dependence of V_{sym} for positive and negative fields. For V_{sym} induced by AMR or thermo-magnetic effects, the expected symmetry is $V_{sym}(+H) = V_{sym}(-H)$, since the imaginary part of the magnetic susceptibility (Eq. 10) is an even function with respect to H. However, for V_{sym} originating from spin pumping, the expected symmetry is $V_{sym}(+H) = -V_{sym}(-H)$ since $V_{SP} \sim \mathbf{z} \times \boldsymbol{\sigma}$. Here $\boldsymbol{\sigma}$ (//$\mathbf{M}$) is the spin polarization vector, and $\mathbf{z}$ is the unit vector in [001] direction[24]. Considering these symmetries, the magnitude of V_{SP} can be accurately determined by

$$V_{SP} = \frac{V_{sym}(-H) - V_{sym}(+H)}{2}. \tag{13}$$

In this case, voltages V_{sym} originating from AMR and thermo-magnetic effects ares cancelled. We have verified this method by using a Py/Pt bilayer where the measured magnetic-field angle dependence of V_{SP} can be well fitted by the inverse spin Hall effect (ISHE) (not shown here), indicating the validity of the analysing method.

Supplementary Figure 10d shows the magnetic-field angle dependence of V_{sym} for a [110]-oriented Fe/GaAs spin pumping device. One can see that $V_{sym}(+H) > V_{sym}(-H)$ for $\varphi_H = 135^{o}$ (perpendicular to the stripe, the perpendicular orientation of external field and stripe is confirmed

by the φ_H dependence of H_R, which is shown in Supplementary Figure 10c), $V_{sym}(+H) = V_{sym}(-H)$ for $\varphi_H = 225^o$ (parallel to the stripe) and $V_{sym}(+H) < V_{sym}(-H)$ $\varphi_H = 315^o$ (perpendicular to the stripe again). This is the expected symmetry of V_{SP} with superimposed signals stemming from AMR and/or thermo-electric effects. The magnitude of V_{SP} is then obtained by Eq. 12, which can be well fitted by the theoretical model of spin pumping (Fig. 5c in the main text). Since the non-magnetic layer is missing and Rashba dominated SOI has been demonstrated, this effect is called spin-galvanic effect, SGE. We have also measured devices with other orientations and from different wafers; all of them show consistent results.

To eliminate the possibility that the observation of SGE is an artefact, we measure a polycrystalline Fe film deposited on an amorphous SiO_x substrate. As shown in Supplementary Figure 11, the voltage goes to zero and no difference between $V_{sym}(+H)$ and $V_{sym}(-H)$ when magnetic field is perpendicular to the stripe. This indicates no existence of SGE for Fe/SiO_x. The angular dependence can be well fitted by AMR effect of Fe. We have also measured Py/SiO_x, and no SGE is observed (Supplementary Figure 12).

Supplementary References

1. Juretschke H. J. Electromagnetic theory of dc effects in ferromagnetic resonance. *J. Appl. Phys.* **31**, 1401-1406 (1960).

2. Hupfauer, T. *et al*. Emergence of spin-orbit fields in magnetotransport of quasi-two-dimensional iron on gallium arsenide. *Nature Commun*. **6,** 7374 (2015).

3. Gilbert, T. L. A phenomenological theory of damping in ferromagnetic materials. *IEEE Trans. Magn.***40,** 3443-3449 (2004).

4. Chen, L., Matsukura, F., & Ohno, H. Direct-current voltages in (Ga,Mn)As structures induced by ferromagnetic resonance. *Nature. Commun.***4,** 2055 (2013).

5. Woltersdorf, G., and Heinrich, B. Two-magnon scattering in a self-assembled nanoscale network

of misfit dislocations. *Phys. Rev.* **B 69,** 184417 (2004).

6. Zakeri, Kh., Lindner, J., Barsukov, I., Meckenstock, R., Farle, M., von Hörsten, U., Wende, H., Keune, W., Rocker, J., Kalarickal, S. S., Lenz, K., Kuch, W., Baberschke, K., Frait, Z. Spin dynamics in Ferromagnets: Gilbert damping and two-magnon scattering. *Phys. Rev.* ***B*** **76,** 104416 (2007).

7. Kurebayashi, H., Skinner, T. D., Khazen, K., Olejník, K., Fang, D., Ciccarelli, C., Campion, R. P., Gallagher, B. L., Fleet, L., Hirohata, A., and Ferguson, A. J. Uniaxial anisotropy of two-magnon scattering in an ultrathin epitaxial Fe layer on GaAs . *Appl. Phys. Lett.* **102,** 062415 (2013).

8. Garate, I. & MacDonald, A. H. Influence of a transport current on magnetic anisotropy in gyrotropic ferromagnets. *Phys. Rev.* **B 80,** 134403 (2009).

9. Garate, I. & Franz, M. Inverse spin-galvanic effect in the interface between a topological insulator and a ferromagnet. *Phys. Rev. Lett.* **104,** 146802 (2010).

10. Kurebayashi, H. *et al*. An antidamping spin-orbit torque originating from the Berry curvature. *Nature Nanotech*. **9**, 211-217 (2014).

11. Qaiumzadeh, A., Duine, R. A., & Titov, M. Spin-orbit torques in two-dimentional Rashba ferromagnets. *Phys. Rev.* **B 92,** 014402 (2015).

12. Matos-Abiague, A. *et al*. Spin-orbit coupling mediated spin torque in a single ferromagnetic layer. *Phys. Rev.* **B 80,** 094424 (2009).

13. Gmitra, M., Matos-Abiague, A., Draxl, C., & Fabian, J. Magnetic control of spin-orbit fields: A first-principle study of Fe/GaAs junctions. *Phys. Rev. Lett.* **111,** 036603 (2013).

14. Fan, X. *et al*. Quantifying interface and bulk contributions to spin-orbit torque in magnetic bilayers. *Nature Commun*. **5,** 3042 (2014).

15. Garello, K. *et al*. Symmetry and magnitude of spin-orbit torques in ferromagnetic

heterostructures. *Nature Nanotech*. **8**, 587-593 (2013).

16. Kim, J. *et al*. Layer thickness dependence of the current-induced effective field vector in Ta | CoFeB | MgO. *Nature Mater*. **12,** 240-245 (2013).

17. Fukami, S. *et al*. Magnetization switching by spin-orbit torque in an antiferromagnet-ferromagnet bilayer system. *Nature Mater*. **15,** 535-541 (2016).

18. Skinner, T. D. *et al*. Complementary spin-Hall and inverse spin-galvanic effect torques in a ferromagnet/semiconductor bilayer. *Nature Commun*. **6,** 6730 (2015).

19. Harder, M., Cao, Z. X., Gui, Y. S., Fan, X. L. & Hu, C.-M. Analysis of the line shape of electrically detected ferromagnetic resonance. *Phys. Rev.* **B 84,** 054423 (2011).

20. Shiomi, Y., Nomura, K., Kajiwara, Y., Eto, K., Novak, M., Segawa, K., Ando, Y., and Saitoh, E. Spin-electricity conversion induced by spin injection into topological insulators. *Phys. Rev. Lett.* **113,** 196601 (2014).

21. Rojas-Sánchez, J. C. *et al*., Spin pumping and inverse spin Hall effect in Platinum: the essential role of spin-memory loss at metallic interfaces. *Phys. Rev. Lett.* **112,** 116602 (2014).

22. Obstbaum, M., Härtinger, M., Bauer, H. G., Meier, T., Swientek, F., Back, C. H., and Woltersdorf, G. Inverse spin Hall effect in Ni81Fe19/normal-metal bilayers. *Phys. Rev.* **B 89,** 060407(R) (2014).

23. Bai, L. H., Feng, Z., Hyde, P., Ding, H. F., and Hu, C. M. Distinguishing spin pumping from spin rectification in a Pt/Py bilayer through angle dependence line shape analysis. *Appl. Phys. Lett.* **102,** 242402 (2013).

24. Bai, L. H., Hyde, P., Gui, Y. S., Hu, C. M., Vlaminck, V., Pearson, J. E., Bader, S. D., and Hoffman, A. Universal method for separating spin pumping from spin rectification voltage of ferromagnetic resonance. *Phys. Rev. Lett.* **111,** 217602 (2013).
